# Factors Associated with Retention to Care in an HIV Clinic in Gabon, Central Africa

**DOI:** 10.1371/journal.pone.0140746

**Published:** 2015-10-16

**Authors:** Saskia Janssen, Rosanne Willemijn Wieten, Sebastiaan Stolp, Anne Lia Cremers, Elie Gide Rossatanga, Kerstin Klipstein-Grobusch, Sabine Belard, Martin Peter Grobusch

**Affiliations:** 1 Center of Tropical Medicine and Travel Medicine, Department of Infectious Diseases, Division of Internal Medicine, Academic Medical Center, University of Amsterdam, Amsterdam, The Netherlands; 2 Centre de Traitement Ambulatoire, Lambaréné, Gabon; 3 Centre de Recherches Médicales de Lambaréné (CERMEL), Lambaréné, Gabon; 4 Institute of Tropical Medicine, University of Tübingen, Tübingen, Germany; 5 Clinical Infectious Diseases Research Initiative, Institute for Infectious Diseases and Molecular Medicine, University of Cape Town, Cape Town, South Africa; 6 Julius Global Health, Julius Center for Health Sciences and Primary Care, University Medical Center Utrecht, Utrecht, The Netherlands; 7 Division of Epidemiology, School of Public Health, Faculty of Health Sciences, University of the Witwatersrand, Johannesburg, South Africa; 8 Department of Pediatric Pneumology and Immunology, Charité-Universitätsmedizin, Berlin, Germany; Curtin University, AUSTRALIA

## Abstract

**Background:**

Retention to HIV care is vital for patients’ survival, to prevent onward transmission and emergence of drug resistance. Travelling to receive care might influence adherence. Data on the functioning of and retention to HIV care in the Central African region are limited.

**Methods:**

This retrospective study reports outcomes and factors associated with retention to HIV care at a primary HIV clinic in Lambaréné, Gabon. Adult patients who presented to this clinic between January 2010 and January 2012 were included. Outcomes were retention in care (defined as documented show-up for clinical visits, regardless of delay) or LTFU (defined as a patient not retained in care; on ART or ART naïve, not returning to care during the study period with a patient delay for scheduled visits of more than 6 months), and mortality. Cox regression analysis was used to assess factors associated with respective outcomes. Qualitative data on reasons for LTFU were obtained from focus-group discussions.

**Results:**

Of 223 patients included, 67.3% were female. The mean age was 40.5 (standard deviation 11.4) years and the median CD4 count 275 (interquartile range 100.5–449.5) cells/μL. In total, 34.1% were lost to follow up and 8.1% died. Documented tuberculosis was associated with increased risk of being LTFU (adjusted hazard ratio (aHR) 1.80, 95% confidence interval (95% CI) 1.05–3.11, P = 0.03), whereas early starting anti-retroviral therapy (ART) was associated with a decreased risk of LTFU (aHR 0.43, 95%CI 0.24–0.76, P = 0.004), as was confirmed by qualitative data.

**Conclusions:**

Retention to HIV care in a primary clinic in Gabon is relatively poor and interventions to address this should be prioritized in the HIV program. Early initiation of ART might improve retention in care.

## Introduction

The HIV epidemic remains one of the biggest challenges to public health worldwide. Although major progress has been made in global access to anti-retroviral treatment (ART), significant challenges remain to assure ART for eligible patients living with HIV. In 2013, 12.9 million patients in low and middle-income countries received ART. Compared to 2011 this was an impressive scaling-up with an additional 1.6 million people on ART [1]. However, according to the 2013 World Health Organization (WHO) guidelines these are only 37% of all patients who qualify for ART [1]. A major challenge is to retain people living with HIV in health care. Adherence to ART is vital for patients’ survival, prevention of emergence of drug resistance and prevention of onward transmission. Various factors have been found to impact negatively on patient retention including stigma, logistic and financial difficulties in transportation to the clinic, younger age [2], lack of education and social support [3,4]. High rates of loss to follow up (LTFU) have been reported for patients not yet started on ART [5]. Pre-ART LTFU is a major concern for HIV care and HIV transmission, but not much data are available on pre-ART LTFU rates and associated risk factors.

Travelling to receive care is increasingly recognized as a barrier to HIV care and both initiation of and adherence to ART [6]. Studies from across Africa have shown that distance to the clinic and difficulty in affording transportation is linked to poor ART adherence and LTFU [6–10]. Several aspects of transportation can be barriers to HIV care. A recent review [4] identified 4 aspects: (a) travel distance, (b) travel time, (c) transportation cost and (d) rural versus urban setting.

Gabon is a middle-income country situated in the dense tropical rainforest of Central Africa. The seroprevalence of HIV in Gabon is estimated at 4.0% [1], but detailed data are missing. Anecdotal data from ongoing clinical trials at the Centre de Recherches Médicales de Lambaréné (CERMEL) in Lambaréné amongst pregnant women in multiple centers suggest a higher HIV prevalence (unpublished data).

The aims of this study were to evaluate treatment outcomes of patients attending the HIV clinic in Lambaréné and to identify clinical, demographic as well as travel-related factors associated with LTFU and mortality.

## Methods

### Study design and setting

The current study was conducted at the HIV clinic in Lambaréné, Gabon (Centre de Traitement Ambulatoire, Lambaréné) and consisted of a quantitative, retrospective part and a qualitative part. For the quantitative, retrospective part, data were collected from patient files and clinical registers between January and April 2013. The focus group discussions for the qualitative part of the study were performed in February and March 2013.

**Fig 1:**
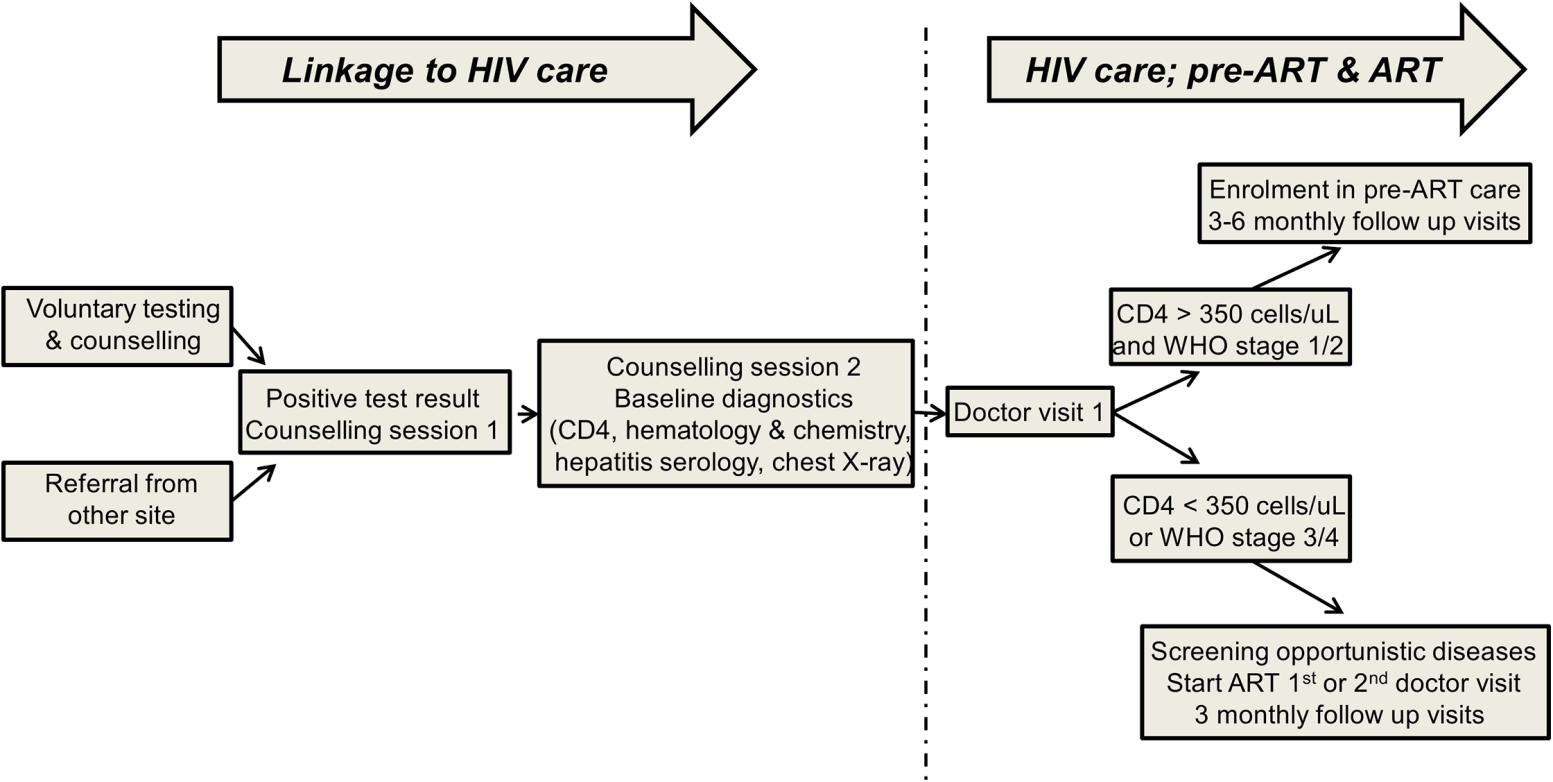
Clinic flow. Fig 1 shows the patient flow upon entry at the clinic.

Lambaréné is a town of 25,000 inhabitants situated within a dense Central African rainforest area in the Moyen Ogooué province. The HIV clinic, founded in 2006, is the main location for HIV care in Lambaréné, apart from the Albert Schweitzer Hospital, where there is also limited capacity to follow up HIV-patients. At the time of the study, the clinic performed voluntary testing and counseling free of charge. If a patient tested positive for HIV, a counsellor would give at least one counselling session before a new patient file was opened and another session before a patient entered HIV care. At every visit to the clinic, patients were additionally seen by a psychologist. ART and treatments for opportunistic infections were provided free of charge. Patients were, however, charged for diagnostic tests and clinical follow up visits, unless patients qualified as ‘social’ cases who could not afford to pay (approximately 25% of patients, personal communication). Whether patients qualified as ‘social’ cases depended on their salary, the chief medical doctor of the clinic making the final decision. ART was initiated in patients who had CD4 counts below 350 cells/μL or were symptomatic with WHO stage 3 or 4 [11]. Patients were regularly (every 3 months) followed up at the clinic, or every 6 months if they were stable in HIV care. Fig 1 shows an overview of the clinic flow upon entry at the clinic.

### Ethical permission

Ethical clearance specifically for this study was obtained from the Institutional Review Board of the CERMEL (Ref # 001/2013). Only information documented for routine patient care was used with no additional patient contacts or study specific inquiries. To that end, informed consent was not needed. Focus-group discussions were held as part of clinical care, and patients’ confidentiality was ensured by anonymizing the information obtained.

### Data collection

#### Quantitative data

Files for all patients who presented to the HIV clinic between January 1^st^ 2010 and January 1^st^ 2012 were reviewed, with only adult patients included in the database. For the final analysis, pregnant women were excluded. Demographic data as well as clinical data on WHO stage, opportunistic diseases, CD4 counts and treatment regimens at presentation and follow up were extracted from patient files and captured in a Microsoft Access (Microsoft Corp., Seattle, WA, USA) database by a research physician (SJ). To determine the influence of travel-related factors on retention in care and outcomes, three variables were derived from patients’ (self-reported) addresses using standardized values: travel distance, time and cost. Values for variables distance and time were derived using a standardized spreadsheet used for clinical trials at CERMEL [12–14] which is based on GPS coordinates. For cost calculation, standardized public transportation costs were used (shared taxis, boats; based on personal communications with clinic staff), as most patients do not own a suitable mean of transport.

Data were supplemented using the HIV clinics’ tuberculosis (TB) register and the register for deceased patients. Follow-up data were recorded until March 1^st^ 2013. Pregnant women were excluded from the analysis, as health care seeking behavior differs significantly when compared to non-pregnant individuals (HIV-testing and if necessary referral is done routinely in pregnancy in Gabon).

#### Qualitative data

In the context of routine clinical care, all adult patients who were re-tracked after being identified as LTFU by this study were invited to participate in focus group discussions (FGDs). Two FGDs consisting of 5–10 adult patients, a counsellor and the research physician (SJ) were conducted in a separate room to ensure confidentiality. In both groups slightly more female patients were present, with the time elapsed since their last clinic visit varying from 6 months to almost 2 years. The subjects of these discussions included reasons for LTFU, stigmatization, and difficulties in adhering to treatment. Information obtained was anonymized and documented by the study physician (SJ).

### Outcomes and variables

Outcomes were retention in care (defined as documented show-up for clinical visits to the doctor (Fig 1), regardless of delay) or LTFU (the opposite of retention to care; defined as a registered patient, on ART or ART naïve, not returning to care during the study period with a patient delay for scheduled visits of more than 6 months), and mortality (defined as documented death in the register for deceased patients). Predefined risk factors were younger age, male sex and rural residence, defined as residence outside of municipal borders of the cities Lambaréné, Libreville or Port Gentil [15–17]. Potential risk factors were determined as CD4 count at entry of HIV care, documented opportunistic infections at entry of HIV care, starting ART at the first or second doctor’s visit and the travel-related factors time, distance and cost to reach the clinic.

### Statistical analysis

The distributions of a priori determined and potential risk factors were compared for patients who were retained in care, LTFU, or deceased, respectively. The χ^2^ test was used for categorical data, the Student’s t-test was used for normally distributed continuous data and non-parametric data were analyzed using the Mann-Whitney U test.

The frequency of missing data was assessed for each variable used in the analysis. The prevalence of missing data for the respective variables was relatively low (max 6.7% for travel distance), and there were no significant differences in missing data in the respective patient groups.

Cox’ proportional hazard analysis was used to determine the association of potential risk factors on loss to follow up and mortality. Main risk factors were identified in univariate analysis. In multivariate analysis, hazard ratios for the main risk factors were adjusted for a priori determined risk factors (age, sex, type of residence). Potential confounders (CD4 count, opportunistic infections, travel-related factors) were assessed for their interaction with the main risk factors. If introduction of a certain factor yielded a change in hazard ratio for one of the main risk factors of >10%, this factor was considered a confounder. All confounders were inserted in the final model, whilst avoiding multi-collinearity. Patients were censored on the day they were last seen in the clinic.

As reported cases of mortality were few, only limited analysis of risk factors for mortality was possible.

Co-infection with TB influences the time of starting ART; patients with TB are less likely to start ART at the first or second clinic visit. Therefore, a subgroup analysis was done excluding TB patients to determine the association of early initiation of ART with respective outcomes.

To assess the differences in retention to pre-ART and ART care, a subgroup analysis was done including 2 patient groups; the group who was eligible for ART and started ART at doctor visit 1 or 2, and a group who was not yet eligible for ART due to a CD4 count > 350 cells/uL. Cox’ proportional hazard analysis was used to determine the respective hazard ratios of being LTFU, adjusted for a priori defined risk factors (age, sex and type of residence). Patients in the pre-ART group starting ART after doctor visit 2 were censored, as well as patients who died within the study period.

Data analysis was done using SPSS version 21 (IBM, Chicago, IL, USA).

Qualitative data of the FGDs was used to enhance understanding of the context and to explain quantitative outcomes. SJ conducted thematic and content analysis.

## Results

A total of 280 patient files were reviewed and captured in the database. After exclusion of patients younger than 18 years and pregnant patients, 223 patient files remained for final analysis.

Baseline characteristics are shown in Table 1. The majority of the cohort was female. The mean age at presentation was 40.5 years (standard deviation (SD) 11.4). The median CD4 count at presentation was 275 cells/uL(interquartile range (IQR) 100.5–449.5 cells/uL). Most patients presented at an early stage of their HIV disease and were classified WHO stage 1 upon presentation. However, a considerable proportion of patients presented at a more advanced stage (37% presenting with WHO stage 3 or 4; Table 1). TB was the most reported opportunistic infection (44/223, 19.7%).

**Table 1:** Baseline characteristics of adult non-pregnant HIV-patients, attending the primary HIV clinic in Lambaréné, Gabon, between January 2010 and January 2012.

Demographics	Data	Total cohort	Retention in care (n = 129)	Lost to follow up (n = 76)	P-values^a^	Deceased (n = 18)	P-values^b^
Female sex (n,%)	223	150 (67.3)	87 (67.4)	48 (63.2)	0.53	15 (83.3)	0.17
Age (years)^c^	223	40.5 (11.4)	41.8 (15)	39.2 (11.6)	0.11	37.1 (11.8)	0.10
Residence (rural) (n,%)	213	90 (42.3)	55 (43.7)	29 (38.7)	0.49	6 (50.0)	0.67
**Travel-related factors**							
Distance to clinic (km)^d^	197	9 (1–473)	11 (1–473)	7 (1–300)	0.63	8 (4–250)	0.86
Time to clinic (hours)^d^	199	0.25 (0.25–8.0)	0.25 (0.25–8.0)	0.25 (0.25–8.0)	0.73	0.25 (0.25–5.0)	0.53
Travel costs (CFA^e^)^d^	199	1500 (200–30000)	1500 (200–30000)	1500 (200–30000)	0.34	1500 (800–14400)	0.66
**Clinical characteristics**							
CD4 count (cells/μL)^f^	211	275 (100.5–449.5)	268 (112–424)	320 (110.5–529.5)	0.06	157 (0–314)	0.25
WHO^g^ stage (n,%)	200					ND	ND
1		92 (46.8)	59 (46.5)	33 (47.1)	0.91		
2		34 (17.0)	24 (18.9)	10 (14.3)			
3		69 (34.5)	40 (31.5)	26 (37.1)			
4		2.2 (2.5)	4 (3.1)	1 (1.4)			
BMI^f^ ^,^ ^h^	151	21.8 (4.5)	22.0 (19.7–24.3)	21.2 (19.0–23.5)	0.36	ND	ND
Hemoglobin (g/dL)^f^	201	10.0 (8.5–11.5)	11.0 (9.5–12.5)	10.0 (8.0–12.0)	0.29	ND	ND
Co-infections (n,%)	223						
*None documented*		140 (62.8)	86 (66.7)	40 (52.6)	0.05	14 (77.8)	0.34
*Oral candidiasis*		20 (9.0)	12 (9.3)	8 (10.5)	0.78	0 (0)	0.36
*Hepatitis B*		14 (14.3)	9 (7.0)	5 (6.6)	0.91	0 (0)	0.60
*Herpes zoster*		17 (7.6)	11 (8.5)	5 (6.6)	0.62	1 (5.6)	0.55
*Tuberculosis*		44 (19.7)	17 (13.2)	23 (30.3)	**0.003**	4 (22.2)	0.29
*Other infections* ^*i*^		8 (3.6)	3 (2.3)	5 (6.6)	0.15	0 (0)	0.68
**Treatment**							
Initiation of ART^j^ at visit 1 or 2 (n,%)	223	139 (62.3)	97 (75.2)	33 (43.4)	**<0.001**	9 (50.0)	**0.03**

Baseline patient characteristics for the total cohort, patients retained in care, patients lost to follow up or deceased patients, respectively.

^a^P-values for comparison of patients lost to follow up to those retained in care.

^b^P-values for comparison of deceased patients to those retained in care.

The χ^2^ test was used for categorical data, the Student’s t-test was used for normally distributed continuous data and non-parametric data were analyzed using the Mann-Whitney U test.

^c^Mean, SD

^d^ Median, range

^e^ Central African Franc (CFA); 1 USD = 550 CFA

^f^Median, IQR

^g^World Health Organization (WHO)

^h^Body Mass Index (BMI)

^I^ Other infections included toxoplasmosis, hepatitis C and pneumonia

^j^Antiretroviral therapy (ART).

Out of 223 non-pregnant patients, 174 (78%) patients were eligible for ART according to WHO guidelines valid during the study period [11]. Ninety-three patients (93/223, 41.7%) started ART during their first physicians’ visit, and another 46 (46/223, 20.6%) patients started ART on the second visit (in total 139/223, 62.3%). The most frequently prescribed drug combination in this cohort was stavudine/lamivudine (64/139, 46.0%), administered together with efavirenz (70/139, 50.4%) or nevirapine (40/139, 28.8%); in accordance with the national treatment guidelines applicable during the study period.

A total of 18/223 (8.1%) patients died during the follow up and a considerable proportion of patients were LTFU (76/223, 34.1%). When compared to patients retained in care, patients who were LTFU started ART less frequently during their first or second clinical visit, had slightly higher CD4 counts and were significantly more often documented to have TB (Table 1). Patients who deceased had a higher prevalence of TB when compared to the patients that were retained in care.

There were no differences among the groups in any travel aspect (distance, time or cost) (Table 1).

The main factor associated with retention to care was initiation of ART at the first or second clinical visit, whereas the main risk factor associated with LTFU was documented TB (Table 2). Even in sub-group analysis excluding patients with documented TB, initiation of ART at the first or second clinical visit was associated with retention to care (adjusted hazard ration (aHR) 0.41, 95% confidence interval (CI) 0.24–0.71, P-value 0.002).

**Table 2:** Factors relating to loss to follow up among adult non-pregnant HIV-patients, attending the primary HIV clinic in Lambaréné, Gabon, between January 2010 and January 2012.

	Univariate analysis			Multivariate analysis		
	HR^a^	95%CI^b^	P-value	aHR^c^	95%CI	P-value
Female sex	0.89	0.56–1.42	0.63	0.82	0.49–1.35	0.43
Age (per 10 years increase)	0.98	0.96–1.00	0.08	0.88	0.70–1.11	0.29
Residence (rural vs. urban)	1.18	0.74–1.87	0.50	0.89	0.56–1.43	0.64
**Travel-related factors**						
Distance to clinic (km)	1.00	(1.00–1.01)	0.72			
Time to clinic (hours)	0.95	(0.85–1.07)	0.42			
Travel costs (per 100 CFA^d^ increase)	1.00	(1.00–1.01)	0.72			
**Clinical characteristics**						
CD4 count (per 50 cells/uL increase)	1.03	1.00–1.07	0.08			
WHO stage^e^	1.06	0.83–1.35	0.66			
BMI^f^	0.97	0.93–1.02	0.25			
Hemoglobin	0.95	0.85–1.05	0.3			
Co-infections						
*None documented*	0.63	0.40–0.98	**0.04**			
*Oral candidiasis*	1.23	0.59–2.57	0.58			
*Hepatitis B*	0.95	0.38–2.35	0.91			
*Herpes zoster*	0.81	0.33–2.02	0.66			
*Tuberculosis*	2.13	1.30–3.48	**0.003**	1.78	1.08–2.94	**0.03**
*Other infections* ^*g*^	1.93	0.78–4.78	0.16			
**Treatment**						
Initiation of ART^h^ at visit 1 or 2	0.36	0.23–0.57	**<0.001**	0.40	0.25–0.64	**<0.001**

Crude and adjusted hazard ratios for associations of respective variables with the outcome loss to follow up.

^a^Hazard Ratio (HR)

^b^Confidence Interval (CI)

^c^Adjusted Hazard Ratio

^d^Central African Franc (CFA); 1 USD = 550 CFA

^e^World Health Organization stage, per stage increase

^f^Body Mass Index (BMI)

^g^Other infections included toxoplasmosis, hepatitis C and pneumonia

^h^Antiretroviral therapy (ART).

**Fig 2:**
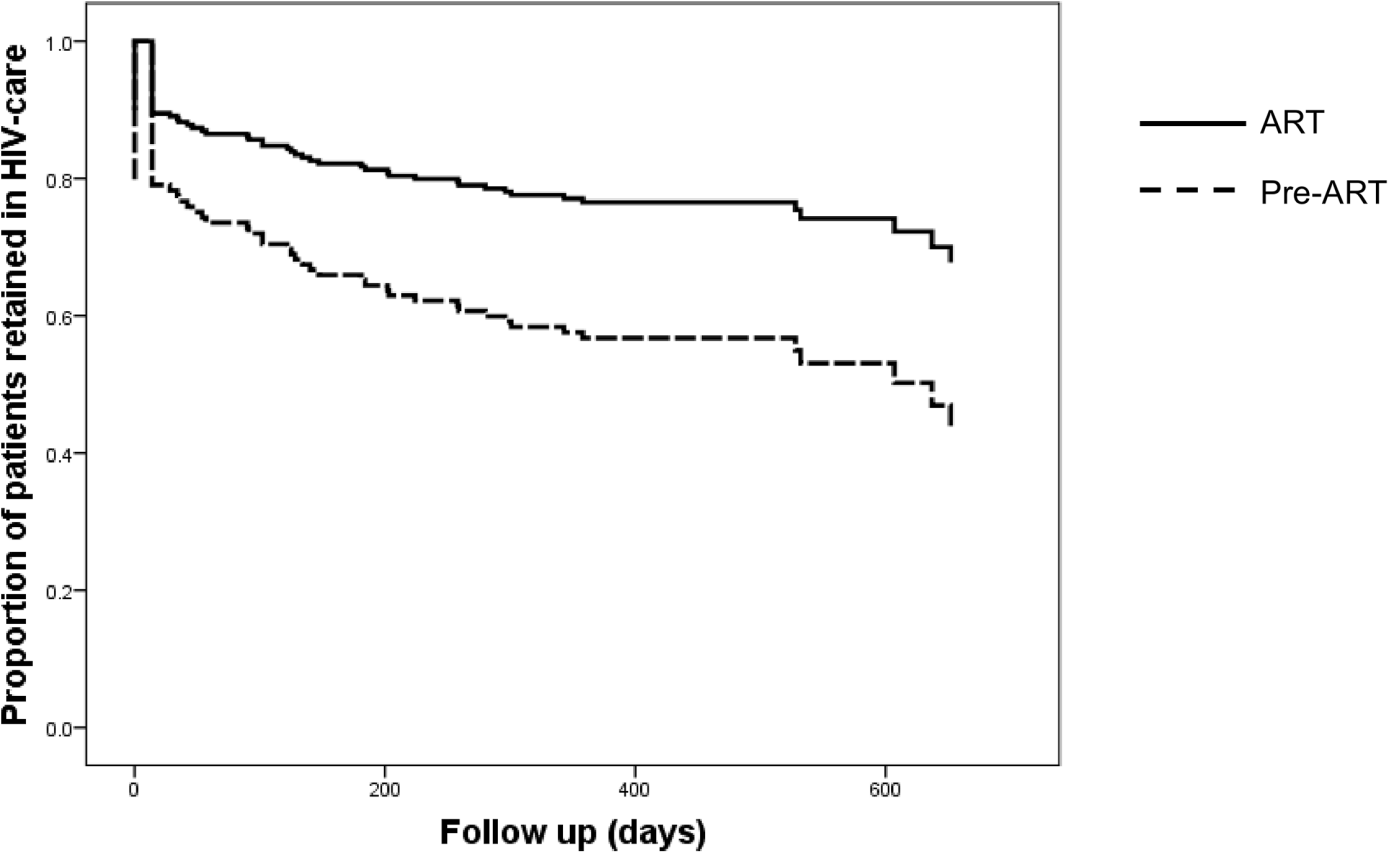
Retention in HIV care. Kaplan Meier curves of retention in care (in days) of patients who started ART at doctor visit 1 or 2 versus patients who were enrolled in pre-ART care. Starting ART was associated with retention in HIV care (aHR 0.47, 95% CI 0.28–0.79, p = 0.004).

Fig 2 shows the Kaplan Meier curves for retention in care comparing the patient groups that started ART at doctor visit 1 or 2 (n/N = 134/197) versus those enrolled in pre-ART care (n/N = 56/197). Median follow up time for the group who started ART was 414 (IQR 286–542) days, whereas the group who was enrolled in pre-ART care was followed up for a median of 358 (IQR 89–627) days, respectively. Starting ART was associated with retention in HIV care (aHR 0.47, 95% CI 0.28–0.79, p = 0.004).

**Fig 3:**
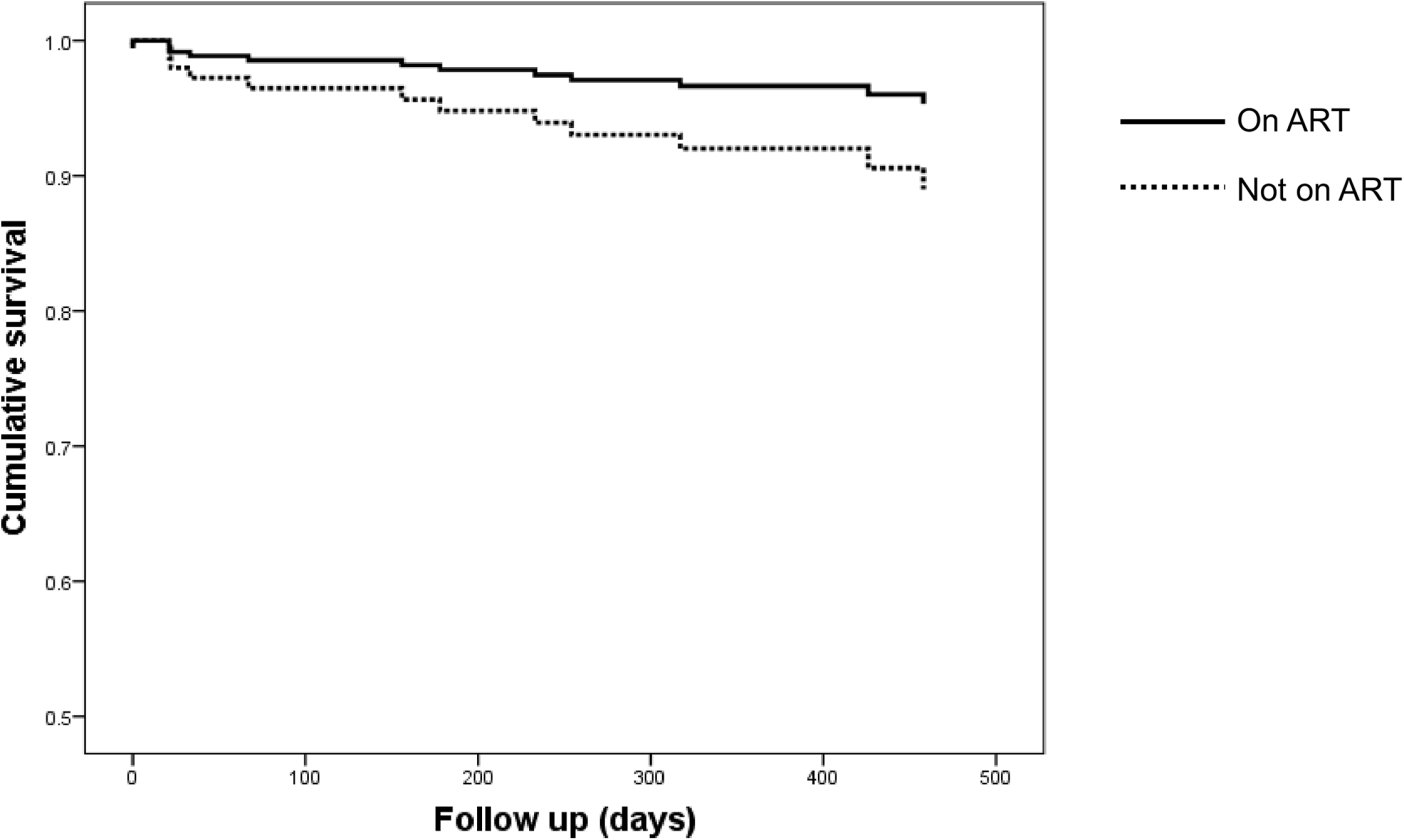
Time to death. Kaplan Meier curves of time to mortality (in days) of patients who started ART versus patients who did not start ART during the first 2 visits to the clinic. There was a trend towards a reduced mortality in patients who started ART.

Fig 3 shows the Kaplan Meier curves for mortality comparing the groups that started ART versus those who did not start. Median time to mortality for the group who started ART was 317 days (range 156–654 days) whereas the group who did not start ART deceased after a median of 67 days (range 0–288 days). Initiation of ART at the 1^st^ or 2^nd^ doctor visit was associated with reduced mortality (cHR 0.38, 95% CI 0.15–0.95, p = 0.04). This did not remain significant in multivariate analysis, probably due to the small sample size (Table 3).

**Table 3:** Factors associated with mortality among adult non-pregnant HIV-patients, attending the primary HIV clinic in Lambaréné, Gabon, between January 2010 and January 2012.

	Univariate analysis			Multivariate analysis		
	HR^a^	95%CI^b^	P-value	aHR^c^	95%CI	P-value
Female sex	2.35	0.68–8.14	0.18	4.47	0.55–36.1	0.16
Age (per 10 years increase)	0.70	0.45–1.08	0.11	0.79	0.46–1.33	0.37
Residence (rural vs. urban)	1.21	0.39–3.71	0.74	1.52	0.48–4.78	0.47
**Travel-related factors**						
Distance to clinic (km)	1.00	0.99–1.01	0.89			
Time to clinic (hours)	0.88	0.62–1.25	0.46			
Travel costs (per 100 CFA^d^ increase)	1.00	0.99–1.01	0.7			
**Clinical characteristics**						
CD4 count (per 50 cells/uL increase)	0.91	0.77–1.08	0.28			
Co-infections						
*None documented*	1.57	0.52–4.79	0.42			
*Oral candidiasis*	0.04	0–100	0.43			
*Hepatitis B*	0.04	0->100	0.45			
*Herpes zoster*	0.69	0.09–5.21	0.72			
*Tuberculosis*	1.91	0.63–5.84	0.25	1.04	0.22–4.79	0.97
*Other infections* ^*e*^	0.05	0->100	0.68			
**Treatment**						
Initiation of ART^f^ at visit 1 or 2	0.38	0.15–0.95	**0.04**	0.37	0.12–1.17	0.09

Crude and adjusted hazard ratios for associations of respective variables with the outcome mortality.

^a^Hazard Ratio (HR)

^b^Confidence Interval (CI)

^c^Adjusted Hazard Ratio

^d^Central African Franc (CFA); 1 USD = 550 CFA

^e^Other infections included toxoplasmosis, hepatitis C and pneumonia

^f^Antiretroviral therapy (ART).

During the FGDs, various factors for LTFU were mentioned, such as adverse effects, long distance to the clinic, lack of time, financial struggles, and stigma. Additionally, two striking problems were identified: Firstly, all patients who were ineligible for ART were disappointed that they did not receive medications at their visits to the clinic. As a consequence, they were reluctant to come to the clinic to merely pay for clinic visits and diagnostic tests. Secondly, patients did not feel welcome at the clinic due to long waiting lines, uncertainty regarding clinic procedures, poor communication and a negative attitude of some health workers towards them.

## Discussion

Understanding the factors associated with failure of retention in HIV care is of vital importance to improve treatment outcomes and reduce HIV transmission.

This study from a primary HIV clinic in a semi-urban setting in the Central African country Gabon shows that a high proportion (34.1%) of all patients was LTFU within a relatively short period of time. Starting ART on the first or second physician contact was associated with a decreased risk of being LTFU, even when excluding TB patients from the analysis, and documented TB at presentation was associated with an increased risk of LTFU. There was no association of travel related factors with baseline characteristics or treatment outcomes. During FGDs, many patients mentioned not starting ART as a factor contributing to their LTFU.

The poor retention in HIV care revealed in this study is comparable to what has been reported from other settings in Africa [18–20]. The main reasons for LTFU mentioned during the FGDs among patients who were tracked and returned to care were frustrations about not starting ART and not feeling supported by the health care staff. These findings exemplify the importance of effective and supportive counselling in HIV care and are in line with previous studies from other settings in Africa reporting that supportive counselling improves adherence to ART [21–24]. A recent systematic review showed that adequate medical and peer support and counselling might improve linkage to HIV care [25]. More studies are needed to investigate how counselling in the Gabonese setting can be improved. Frustrations about not starting ART might be compensated by introduction of food incentives or medical incentives such as co-trimoxazole preventive therapy [25], although this evidence results from few observational studies, and it is unclear whether this would be superior to adequate counselling alone.

A risk factor for LTFU was TB co-infection. There are no quantitative data on the reasons for loss to follow up in this group; however, this may in part be caused by a high mortality in this group of patients. Patients may experience more adverse drug effects, which could explain their renunciation from care. Experience of adverse effects has been reported to impede adherence to TB treatment in several settings [26–28]. As there is still a significant impact of traditional health care in Gabon, a third explanation is the health care seeking behavior of (HIV- or TB-infected) patients in Gabon; many patients still seek care outside the public health care setting [29].

Starting ART on the first or second physician contact was the main factor associated with a decreased risk of being LTFU. This is in line with previous studies from sub-Saharan Africa, showing that patients eligible for ART were less likely to become lost to programme [30]. One other explanation for the high proportion of patients LTFU who did not start ART at the first or second doctor’s contact, is that they might have been asymptomatic and thus less motivated to adhere to care. However, higher CD4 counts were not associated with an increased risk for being lost to follow up (Table 2), and median CD4 counts for patients retained in care versus patients LTFU did not differ substantially (Table 1). Not having symptoms was not mentioned during FGDs as a factor contributing to not adhering to the clinic.

The lack of associations of travel-related factors on outcomes assessed may be explained by the different reasons for travelling to this particular clinic. We learned from clinical experience that a subgroup of patients presenting at the HIV clinic in Lambaréné preferred to travel long distances to attend this HIV clinic to avoid stigmatization in their usual environment of daily living. In this study, 17/223 patients (7.6%) resided in a town with a primary HIV-clinic, but instead they chose to travel to the local HIV clinic in Lambaréné. Possibly, these patients were from a better socio-economic background and were able to pay the cost of transport to go this clinic, instead of the clinic that was closer to their homes. Purposefully travelling long distances to receive HIV care, as seen in Gabon, differs from the travel characteristics previously described in resource poor settings [6–10] where in most cases patients were forced to travel due to long distances from remote areas to the closest HIV clinic. These differences between our study and previous publications from other settings highlight the importance of more studies on health care seeking behavior and adherence of HIV-infected patients to HIV care in in Gabon in order to improve retention to HIV care.

Mortality in this cohort was 8.1%. As shown in Fig 3, there was a trend towards lower mortality in patients who started ART at the first or second physician’s contact, which was significant in univariate analysis (Fig 3, Table 3). The fact that this finding did not reach statistical significance in multivariate analysis is most probably caused by a lack of statistical power due to low patient numbers. The study population consisted of patients residing in different provinces in Gabon and HIV clinics in the country are managed at a national level. The majority of the provinces of Gabon consist of rural areas, similar to the setting of Lambaréné. Therefore, findings reported here may well be generalized to the rest of Gabon and possibly beyond.

There are several limitations of this study. First, the retrospective nature of this study causes important gaps in data completeness. For example, there were not enough data on employment to allow inclusion of socio-economic status as a variable. However, completeness of data for the variables included in this analysis was relatively good. Second, many patients who were LTFU could not be tracked, due to incorrect contact numbers, or possibly death. The lack of integrated health information systems in Gabon disables accurate information gathering on definite LTFU, migration to other clinics or mortality. FGDs were held in the context of clinical care as part of counselling. More extensive qualitative research on reasons for LTFU would offer more insights in the influence of other factors, such as socio-economic factors or traditional medicine. Last, the study size was limited, however, in view of the size of the population of Gabon (2 million people) and the moderate HIV prevalence, the size of this study is considerable. In particular, absolute numbers of patients who deceased during the study period were few, and limited numbers in the analysis may have masked associations of variables assessed with mortality.

## Conclusion

Retention in pre-ART care is vital to allow for integration of prevention strategies in HIV care, to reduce HIV transmission, and for timely initiation of ART [31]. Patients who are lost to pre-ART care might continue to spread the virus and may present later with an advanced symptomatic stage of disease.

Over the last years, guidelines for initiation of ART have been adapted and the CD4 threshold under which initiation of ART is recommended has increased. WHO guidelines now recommend initiation of ART in all individuals with CD4 counts below 500 cells/μL [32], whereas guidelines in the United States of America recommend treatment irrespective of CD4 count [33]. Early initiation of ART has been reported to reduce mortality and ongoing transmission [34,35]. The findings of this study are supportive for early initiation of ART, in order to improve retention to HIV care and thereby reduce transmission. Early initiation of ART should be combined with intensive and supportive counselling, as personal concerns of patients might form barriers to adherence [36]. Caution is warranted with early initiation of ART and more prospective studies on adherence and retention in care are needed, as initiating ART in patients who are insufficiently motivated may be detrimental.
